# Pleistocene island connectivity did not enhance dispersal or impact population size change in Galápagos geckos

**DOI:** 10.1098/rspb.2025.0746

**Published:** 2025-05-28

**Authors:** Edward A. Myers, Rayna C. Bell, Isaac Overcast, Jaime A. Chaves, Omar Torres-Carvajal

**Affiliations:** ^1^ Department of Herpetology, California Academy of Sciences, San Francisco, CA, USA; ^2^ Institute for Biodiversity Science and Sustainability, California Academy of Sciences, San Francisco, CA, USA; ^3^ Colegio de Ciencias Biológicas y Ambientales, Universidad San Francisco de Quito, Quito, Pichincha, Ecuador; ^4^ Biology, San Francisco State University, San Francisco, CA, USA; ^5^ Galapagos Science Center, Universidad San Francisco de Quito USFQ and University of North Carolina-Chapel Hill, San Cristobal, Galapagos, Ecuador; ^6^ Museo de Zoología QCAZ, Biología, Pontificia Universidad Católica del Ecuador, Quito, Ecuador

**Keywords:** oceanic island, *Phyllodactylus*, Galápagos, Pleistocene, demography, introgression

## Abstract

Patterns of biodiversity on remote archipelagos are largely shaped by intra-archipelago colonization followed by *in situ* diversification. Pleistocene sea-level fluctuations purportedly enhanced gene flow among terrestrial organisms by increasing connectivity during periods of lower sea level. Furthermore, changes in sea-level are hypothesized to impact population sizes as a result of fluctuations in island sizes. Here, we used genomic data to test the role of Pleistocene island connectivity on the diversification and demographics of leaf-toed geckos (*Phyllodactylus*) endemic to the Galápagos. Consistent with previous studies, we found that present diversity of Galápagos *Phyllodactylus* stems from three independent dispersal events. Contrary to the hypothesis of Pleistocene-driven diversification, we found no correspondence between lineage divergence and island connectivity. Furthermore, we found no evidence of introgression; demographic modelling indicated that all species increased rapidly in effective population size in the period 20–150 ka, and these inferred demographic expansions were largely asynchronous and apparently unassociated with species or island age. Collectively, these results indicate that more complex abiotic and/or biotic factors may better explain the recent demographic history of *Phyllodactylus* and underscore the need for additional population genomic studies of terrestrial taxa to understand the impact of past climate cycles on Galápagos island communities.

## 1. Introduction

Immigration, *in situ* diversification and extinction are the major biological processes that shape patterns of biodiversity on islands [1,2]. Island biogeography models suggest that within island and within archipelago diversification plays an outsized role in generating unique biodiversity on remote islands where dispersal from continental sources is low [3,4]. Such radiations are typical for many taxa in remote archipelagos such as the Galápagos and Hawaii [5–8]; however, not all species that reach these islands diversify. For instance, Ali & Meiri [9] showed that for many non-volant reptiles on volcanic islands, immigration and anagenesis are the major processes that contribute to the accumulation of biodiversity. Importantly, Ali & Meiri [9] distinguished two types of reptile assemblages within archipelagos worldwide. The first contains many species-poor clades that are descendants of disparate lineages of external immigrants (e.g. Lesser Antilles, Comoros, Gulf of Guinea), whereas the second type is composed of fewer clades, some species-rich, which were built largely through anagenesis following intra-archipelago colonization (e.g. Galápagos, Canaries). Identifying the geological context of intra-archipelago colonization and anagenesis is thus key to understanding the assembly and growth of biodiversity in remote archipelagos.

**Figure 1.**
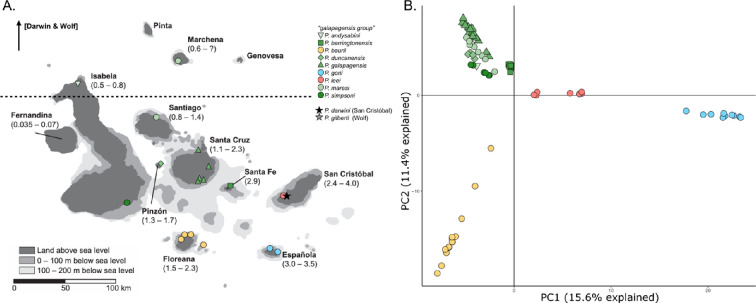
(A) Map of the primary islands of Galápagos and sampling localities of leaf-toed geckos (*Phyllodactylus* spp). Approximate ages in millions of years are indicated for islands on which leaf-toed geckos occur. Bathymetric contour lines delimit depths up to 200 m below sea level. The islands of Santa Fe, Santa Cruz, Santiago, Pinzón, Isabela and Fernandina form an island complex in which presently isolated landmasses have been connected during periods of lower sea level. The dashed line indicates the equator. Island age estimates from Geist *et al*. [14]. (B) Principal component analysis of unlinked SNPs from the *galapagensis* clade. Symbols and colors follow the same legend as the map on the left.

The Galápagos archipelago in the Tropical Eastern Pacific is located approximately 1000 km west of South America and hosts exceptionally unique biodiversity, with some lineages that have diversified extensively and others that have not [8]. Organisms with passive dispersal (like terrestrial vertebrates) are expected to colonize geographically remote islands sequentially, starting from the oldest to the youngest [10]. Strikingly, the oldest extant lineages of most terrestrial reptiles in the Galápagos, as well as other land organisms like snails [11], occur on the oldest islands (San Cristóbal or Española), and their colonization of the archipelago generally follows an old-to-young island pattern [12]. Despite their limited over-water dispersal abilities, it has been generally assumed that terrestrial reptiles have radiated within the archipelago by overseas dispersal from one island to another [13–16]. Yet, many islands in this archipelago have periodically been connected by exposed land bridges when sea levels were lower during the Pleistocene (figure 1). Therefore, as a consequence of more land being exposed above water, the total area of the Galápagos Islands was much greater during Pleistocene glaciations than it is today [14], and terrestrial organisms may have dispersed across the archipelago during these periods of greater connectivity [17]. These past intermittent connections among islands and islets that are presently separated by salt water may have further shaped population structure and diversification of Galápagos terrestrial biota by allowing for introgression among populations from different islands when sea levels retreated, followed by evolution in isolation as sea levels rose [14,17]. Such introgression can result in adaptive evolution and potentially drive species diversification [18–20]. Introgressive hybridization has also occurred in cases of long-distance dispersal, for example, inter-island dispersal of a single Galápagos finch resulted in hybridization with the local finch species and the formation of a new hybrid lineage that is reproductively isolated from its parental species through mate recognition [21]. In contrast, inter-island dispersal within Galápagos tortoises has been associated with the breakdown of assortative mating, resulting in lineage fusion [22]. Thus, the connectivity of islands and organismal dispersal between them may have played a particularly important role in the expansion and diversification of terrestrial species on the largest and most interconnected island complex (Santa Fe, Santa Cruz, Pinzón, Santiago, Isabela, Fernandina; figure 1) but this hypothesis has not yet been tested for Galápagos taxa with empirical data.

The endemic leaf-toed geckos are unique among Galápagos organisms in that they originated from three asynchronous colonization events from South America; two of them (approx. 0.69 Ma and approx. 3.03 Ma) both led to an endemic species via anagenesis (*Phyllodactylus giberti* on Wolf Island and *P. darwini* on San Cristóbal Island, respectively), while the third led to a radiation starting at approximately 5.49 Ma that gave rise to nine species [23,24]. This third and oldest radiation, herein referred to as the ‘*galapagensis* clade’ (*P. andysabini*, *P. barringtonensis*, *P. baurii*, *P. duncanensis*, *P. galapagensis*, *P. gorii*, *P. leei*, *P. maresi* and *P. simpsoni*), occurs in the largest and most interconnected island complex (figure 1) and most islands harbor a single endemic species, except for San Cristóbal (*P. darwini* and *P. leei*) and Isabela (*P. andysabini* and *P. simpsoni*). Most of the leaf-toed geckos inhabit lowland, dry coastal habitats and therefore fluctuating sea levels throughout the Pleistocene would have shifted the size and distribution of their habitats. At present, island sizes are smaller than they were at the Last Glacial Maximum (LGM) and thus gecko populations across all islands may have experienced a recent and concurrent decrease in population size in response to this shared palaeo-climatic history (e.g [25]). Consequently, leaf-toed geckos present an ideal radiation in which to understand how dispersal and vicariance across a dynamic landscape contributed to expansion and diversification within the archipelago.

In this study, we investigated the role of connectivity within island banks during the Pleistocene on the diversification of Galápagos endemic leaf-toed geckos. To this end, we first estimated evolutionary relationships among all leaf-toed geckos using a phylogenomic approach to evaluate current diversity and distributions of species across sampled islands. Second, we characterized genetic structure and introgression within the *galapagensis* clade among islands to test the hypothesis that geckos dispersed across exposed land bridges when islands were connected during Pleistocene glaciations. Third, we estimated divergence times to test the hypothesis that there is correspondence between lineage splitting, island ages and Pleistocene palaeogeographical models. Finally, we inferred changes in effective population size through time to test the hypothesis that post-Pleistocene decrease in island area had an effect on leaf-toed gecko populations and that there is a signal of shared demographic changes across species within the *galapagensis* clade.

## 2. Methods

### (a) Sample collection

We sampled 90 individuals representing all 11 currently recognized species of *Phyllodactylus* in the Galápagos, as well as six individuals of two mainland *Phyllodactylus* species (electronic supplementary material, table S1). Galápagos samples were collected from 13 different islands and islets (figure 1; electronic supplementary material, table S1). All samples were obtained from the genomic collection of Museo de Zoología (QCAZ) at Pontificia Universidad Católica del Ecuador.

### (b) DNA sequencing + ddRAD bioinformatics

We extracted DNA from tissue samples and submitted DNA to the University of Wisconsin-Madison Biotechnology Center for genotyping-by-sequencing (GBS) services using a dual enzyme DNA digest protocol. Sequencing was performed using paired read, 150 bp sequencing on a partial lane of an Illumina NovaSeq.

We used ipyrad v0.9.59 [26] to demultiplex Illumina reads and assemble loci. We constructed two datasets, one that included all *Phyllodactylus* species found across the Galápagos and the mainland outgroups, and another that included only the core *galapagensis* clade, a nine species radiation [23]. For details on GBS library prep and bioinformatics, see the supplemental materials.

### (c) Phylogenetic analyses and divergence dating of Galápagos geckos

To estimate phylogenetic relationships, we generated two different concatenated gene trees in IQtree v2.2.0 [27,28]: one with the full dataset consisting of 96 individuals and a second with only the *galapagensis* clade. We also estimated an unrooted species tree using SVDquartets [29]. For this analysis, we included all sequenced *Phyllodactylus* individuals, grouping them by species. Lastly, we used Beast v2.6.7 to estimate divergence times [30]. First, we selected one individual per species with the least amount of missing data and concatenated all GBS loci from the full dataset (a total of 3 038 637 bases). Using this matrix, we applied a clock rate of 1.1 × 10^–9^ mutations/site/year as the per lineage mutation rate [31], used the GTR + G +I model of nucleotide substitution, and applied a birth-death tree prior. For further details on phylogenetic analyses, see the supplemental materials.

### (d) Population structure and introgression in the galapagensis clade

We used principal component analysis (PCA) to assess whether individuals clustered within the islands they were collected from and the species to which they were identified using the *adegenet* library [32] in R. We also used a model-based approach to assess potential shared ancestry between species using the maximum likelihood-based approach ADMIXTURE [33]. See the supplemental materials for details on these analyses.

To assess whether there is a signal of past introgression between the Galápagos *Phyllodactylus* species, we used the multispecies network coalescent approach SNaQ implemented in the Julia package *PhyloNetworks* [34]. Details of this analysis are provided in the supplemental materials.

### (e) Population size change since the Last Glacial Maximum

To infer past changes in effective population size of each species, we used Stairway Plot v2.1 [35]. This method uses a multi-epoch model and calculates a composite likelihood given a site frequency spectrum (SFS) [36]. We estimated the folded SFS for each species from vcf format files using *easySFS* (https://github.com/isaacovercast/easySFS). We used the down projection method to sample a smaller number of individuals from the dataset, averaging over resampling schemes to construct a complete data matrix. The number of haploid samples and retained SNPs after down projection are listed in electronic supplementary material, table S2. *Phyllodactylus andysabini* was excluded from this analysis as we sampled only one individual from this species. *Phyllodactylus maresi* is presently considered a single species but occurs on two separate islands (Santiago and Marchena) with substantial genetic divergence between the populations (see results). Consequently, we ran separate demographic models for each population. We ran Stairway Plot to fit the multi-epoch demographic model to each species, assuming a 1-year generation length, a mutation rate of 2.2 × 10^–9^ [31] and 200 bootstrap replicates to infer a 95% confidence interval. Due to restrictions in collecting within the Galápagos archipelago, some of our species have limited numbers of individuals; however, it has been demonstrated that the accuracy of SFS-based methods is contingent on the number of segregating sites and not the number of individuals included in these analyses [37]. Therefore, we follow the suggestions of Noskova *et al*. [38] in considering the limitations of such approaches and implement simplified demographic models that consider changes in effective population size only.

### (f) Concordance in historical demographic trajectories

After inferring population size change histories within each species, we estimated the degree of concordance in the timing of size change across the core *galapagensis* clade. To achieve this, we deployed a hierarchical simulation-based machine learning approach implemented in the Phylogeographic Temporal Analysis (PTA) package (https://github.com/isaacovercast/PTA). PTA adopts a model-based statistical comparative phylogeographic approach [39,40] for inferring the proportion of species undergoing synchronous expansion (ζ) and the timing of the co-expansion pulse (τ_s_). The hyperparameter ζ can take values from 0 to 1, with 0 indicating asynchronous demographic histories, and 1 indicating fully synchronous demographic histories. The PTA workflow involves three steps: (i) parametrizing the model and running simulations; (ii) training and validating a machine learning model; and (iii) estimating parameters for the empirical data and assessing goodness of fit. We further adopted a nested approach to infer synchrony in co-expansion. For PTA, the dimension of each SFS for all species needs to be identical, which allows for the exchangeability among SFS bins, an assumption of the model [41,42]. We used *easySFS* to down-project all species to eight haploid samples to maximize the number of retained SNPs in each taxon. First, we fit a global model for all species included in the Stairway Plot analyses (i.e. excluding *P. andysabini*) sampling from a uniform prior on τ_s_ (i.e. approx. U(40−200 ka)). Next, we fitted submodels to reduced species sets that were inferred by Stairway Plot to have expanded either recently (*P. gorii*, *P. barringtonensis*, *P. galapagensis*, *P. baurii*) or more distantly (*P. simpsoni*, *P. maresi* Santiago, *P. leei*, *P. duncanensis*) in the past, with priors on τ_s_ constrained approximately to the Last Glacial Period (LGP; approx. 40–100 ka) and the Penultimate Glacial Period (PGP; approx. 100–200 ka), respectively. As it was estimated to have expanded much more recently than any other species (approx. 20 ka), *P. maresi* (Marchena) was not included in the nested co-expansion analysis.

## 3. Results

We obtained a total of 321 million sequence reads with an average of 3.35 million reads per sample. The dataset across all 96 samples, when allowing for 50% missing data per sample, contained 14 448 GBS loci, a total of 3.04 million bases, 157 294 parsimony-informative sites, and 300 268 total variable sites. The *galapagensis* clade-only dataset resulted in 24 103 loci, totaling 5.01 million bases, with 176 342 parsimony-informative sites and 394 026 variable sites. Within the *galapagensis* dataset missing data on a per sample basis ranged from 15.9 to 78.1% (mean = 34.6%; see electronic supplementary material, figure S1). For the full dataset, missing data per sample ranged from 12.1 to 89.6% (mean = 38.9%; see electronic supplementary material, figure S1). Demultiplexed fastq files are accessioned in NCBI (BioProject number PRJNA1088269).

### (a) Phylogenomics and divergence dating of Galápagos geckos supports three dispersal events

**Figure 2.**
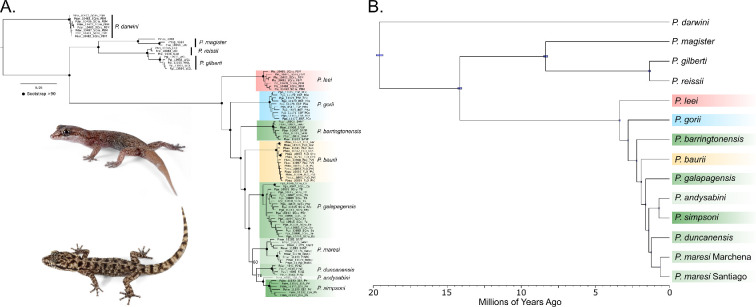
Phylogenetic relationships within the Galápagos Island *Phyllodactylus* radiation. (A) Maximum likelihood gene tree inferred from concatenated unlinked SNPs. Black circles at nodes represent bootstrap support values greater than 90. (B) Divergence times estimated for all *Phyllodactylus* geckos found within the Galápagos Islands as well as two closely related mainland species. Images of *P. leei* (top) and *P. galapagensis* (bottom) were taken from Bioweb.bio.

The concatenated gene trees for the full dataset and the *galapagensis* clade had similar, well-supported topologies where all species form monophyletic groups (figure 2; electronic supplementary material, figure S2). *Phyllodactylus andysabini* was only represented by a single sample and was strongly supported as the sister to *P. simpsoni* (*n* = 6). The SVDquartets species tree showed similar relationships to the concatenated analyses (electronic supplementary material, figure S3) with slight differences in the relationships among *P. duncanensis*, *P. maresi* and *P. simpsoni*. The SVDquartets species tree was generally well supported (Bootstrap [BS] ≥99), with the exception of the node uniting *P. maresi* with (*P. simpsoni*, *P. andysabini*) (BS = 89).

The phylogenetic and divergence dating analysis in BEAST reached stationarity with all ESS values>200 and all nodes being strongly supported (PP = 1.0; figure 2). This tree was similar in topology to the other phylogenetic analyses, with the exception of the relationships among *P. duncanensis*, *P. maresi* and *P. simpsoni*. The estimated node age for the *galapagensis* clade was 3.36 Ma (95% HPD = 3.31–3.39 Ma) and the stem age of this group was estimated to be 14.15 Ma (14.01–14.39 Ma). The estimated divergence times between the two *P. maresi* populations (i.e. Santiago and Marchena islands) was 607 ka (590–626 ka). The divergence time between *P. gilberti* and its mainland sister taxon was estimated to be 1.34 Ma (1.28 – 1.41 Ma) and the estimated divergence among all *Phyllodactylus* geckos distributed in the Galápagos Islands along with their mainland relatives was 19.59 Ma (19.35 – 19.8 Ma). A summary of the estimated age for each species along with island ages is presented in table 1.

**Table 1.** Lineage and island age estimates (Ma) of species of *Phyllodactylus* in the *galapagensis* clade. Island age estimates from Geist *et al*. [14]. Taxa are sorted by age, from oldest to youngest. LGP— Last Glacial Period. PGP— Penultimate Glacial Period. LGM—Last Glacial Maximum. Asterisk indicates asynchronous expansion time.

Species	Species age mean (95% HPD)	Island	Island age (min–max)	Island area (km^2^)	Island max elevation (m)	Co-expansion epoch (estimated timing)
*P. leei*	3.36 (3.32, 3.40)	San Cristóbal	2.4−4.0	558	713	PGP (179 ka)
*P. gorii*	2.79 (2.76, 2.82)	Española	3.0−3.5	60	206	LGP*
*P. barringtonensis*	2.21 (2.18, 2.24)	Santa Fe	2.9−2.9	24	259	LGP (64 ka)
*P. baurii*	1.90 (1.87, 1.92)	Floreana	1.5−2.3	173	640	LGP (64 ka)
*P. galapagensis*	1.59 (1.57, 1.61)	Santa Cruz	1.1−2.3	986	864	LGP (64ka)
*P. duncanensis*	1.21 (1.19, 1.23)	Pinzón	1.3−1.7	18	458	PGP (179 ka)
*P. andysabini*	0.70 (0.68, 0.72)	Isabela	0.5−0.8	4586	1707	NA
*P. simpsoni*	0.70 (0.68, 0.72)	Isabela	0.5−0.8	4586	1707	PGP (179 ka)
*P. maresi*	0.61 (0.59, 0.63)	Santiago	0.6−1.4	585	907	PGP (179 ka)
*P. maresi*	0.61 (0.59, 0.63)	Marchena	0.6—?	130	343	LGM*

### (b) No evidence of introgression in the *galapagensis* clade

The PCA plot of the *galapagensis* clade dataset showed distinct clusters for all species with many of the more recently diverged species grouping together in PC space (figure 1). PCA based on more stringent filters of missing data resulted in similar clustering of individual samples (electronic supplementary material, figure S4). The ADMIXTURE model with all species in the *galapagensis* clade had a best-fit K = 7. This K-value grouped *P. duncanensis*, *P. simpsoni* and *P. andysabini* into one cluster while finding all other named species as distinct clusters (electronic supplementary material, figure S5). This analysis did not find any admixed ancestries among the named species. When assessing genetic structure among *P. duncanensis*, *P. simpsoni* and *P. andysabini*, a model of K = 2 separated *P. duncanensis* from *P. simpsoni* and *P. andysabini* with no admixture (electronic supplementary material, figure S6). The multispecies coalescent network-based approach SNaQ indicated that a strictly bifurcating history, with no instances of reticulation, was the best-fit model for the *P. galapagensis* clade (electronic supplementary material, figure S7).

### (c) Historical population demography is not correlated with Pleistocene climate fluctuations

**Figure 3.**
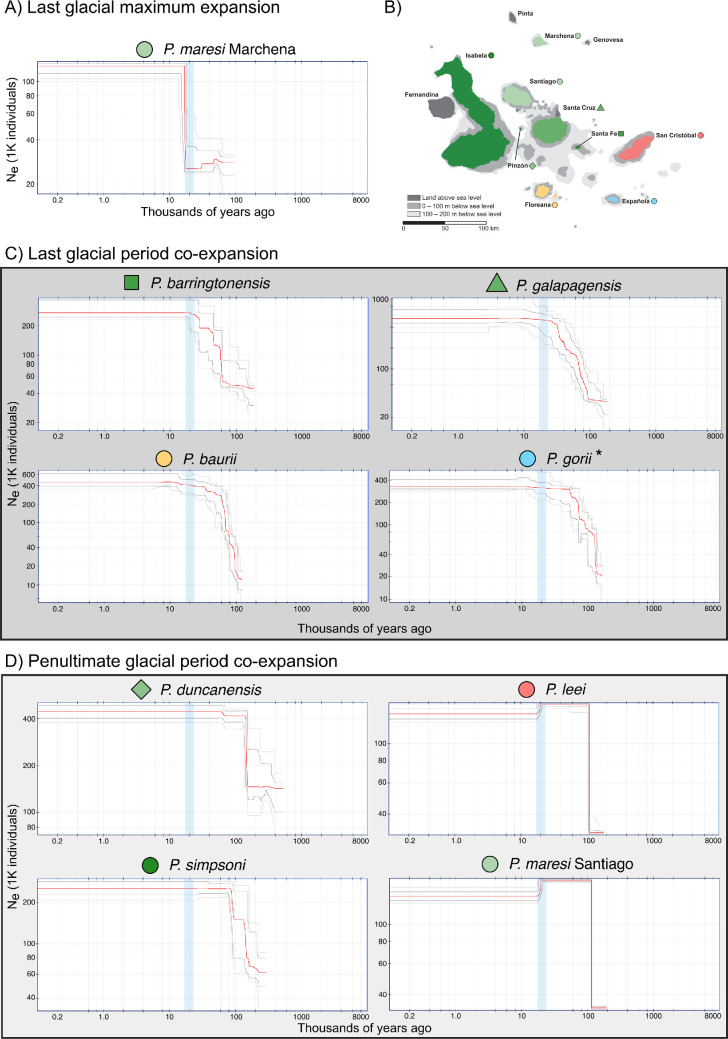
Demographic histories of the *galapagensis* clade inferred using Stairway Plot. The red line in each plot represents the median inferred effective population size through time, light grey lines represent the 95% confidence intervals and darker grey lines the 75% confidence intervals. Vertical blue bars on each plot denote the LGM. (A) The demographic history of the *P. maresi* population on Marchena island did not have a similar timing of change compared to other species in this radiation. (B) Map of the Galápagos islands color coded to indicate the geographic distribution of each species. Panels (C) and (D) represent the sets of taxa that were analysed together in the nested Phylogeographic Temporal Analysis to test for co-expanding species. (C) Species found to be co-expanding during the Last Glacial Period, note that *P. gorii* was found to have an asynchronous history compared to these three other species. (D) The species supported to have synchronous population expansions at the penultimate glacial cycle.

The Stairway Plot analyses demonstrated that all species have increased in effective population size with no indications of population size decreases since the LGM (figure 3). These increases in population size were inferred to have occurred between 20 and 150 ka. Additionally, these increases in size occurred rapidly and quickly reached the current estimated size. Current effective population sizes are estimated to be between approximately 104 000 and 334 000 individuals.

### (d) Asynchrony in historical demographic trajectories

Validation of the PTA simulation-based machine learning approach demonstrated reasonable accuracy to infer the proportion of co-expanding taxa (ζ) and timing of co-expansion (τ_s_; electronic supplementary material figures S8 and S9). The global PTA model fit with the full *galapagensis* clade dataset found a mode estimate for ζ of 0.33 (electronic supplementary material, figure S10; prediction probability 0.24) indicating synchronous expansion of three out of nine species. There was reduced and approximately equal prediction probability for ζ of 0.2 and 0.4 (0.18 and 0.17, respectively) and little support for ζ less than 0.2 or greater than 0.4, suggesting that partial synchrony in co-expansion was the best fit model (between two and four species co-expanding synchronously). The timing of co-expansion was estimated to be approximately 161 ka, though the prediction interval (51–196 ka 95% PI) was wide. Nested analysis of the recent (LGP: *P. barringtonensis* —Santa Fe, *P. baurii* —Floreana, *P. galapagensis*—Santa Cruz, *P. gorii*—Española) and distant (PGP: *P. duncanensis*—Pinzón, *P. leei*—San Cristóbal, *P. maresi* - Santiago, *P. simpsoni*—Isabela) expansion groups independently found strong support for ζ = 0.75 (electronic supplementary material, figure S11; three co-expanding species) in the LGP group and ζ = 1 (electronic supplementary material, figure S12; synchronous co-expansion of all four species) in the PGP group. Estimated τ_s_ for LGP (64 ka) and PGP (179 ka) were both bracketed by narrower prediction intervals (42–79 ka and 168–193 ka, respectively), indicating there was sufficient information in the data to estimate these parameters with some precision.

## 4. Discussion

### (a) Galápagos geckos are the result of three asynchronous dispersal events from South America

Most of the endemic land reptile groups (tortoises, lizards and snakes) in the Galápagos are the product of intra-archipelago radiations, each descended from a single colonization. Only lava lizards (*Microlophus*) originated from two colonization events leading to radiations at approximately 1.4 and 0.4 Ma that resulted in eight and two species, respectively [13,24,43]. Remarkably, leaf-toed geckos are unique among Galápagos endemic land vertebrates in that their present diversity stems from three independent, asynchronous colonization events. This scenario has been proposed in previous studies with more limited genetic data [24,44] and is fully supported by the phylogenomic dataset presented here (figure 2). Furthermore, our divergence time estimates for the *galapagensis* clade (node age = 3.36 Ma [95% HPD = 3.31 – 3.39 Ma]; stem age = 14.15 Ma [14.01–14.39 Ma]) are similar to previous estimates derived from a more limited dataset (node age = 5.49 Ma [95% HPD = 4.54–6.39 Ma]; stem age = 13.8 Ma [7.92–20.21 Ma] [23]). Thus, our results support the hypothesis that together with the tortoises, leaf-toed geckos could have colonized the Galápagos as early as in the Middle Miocene (15.97−11.63 Ma), which largely predates the age of present islands (3–4 Ma). Multiple lines of geological evidence support the existence of subaerial islands in the Middle Miocene and Late Miocene [45–47], which could have hosted the ancestors of present-day Galápagos land vertebrates. However, our divergence time estimates also suggest that the intra-archipelago radiation of the *galapagensis* clade happened when present islands had emerged, as most species age estimates fall within the emergence estimates of the islands where they occur (table 1). Previous studies based on more limited datasets proposed both *P. leei* and *P. gorii*, which occur on the oldest islands—San Cristóbal and Española, respectively, as candidates for oldest lineage within the *galapagensis* clade [23,24,44]. This pattern is consistent across several distantly related groups of Galápagos organisms including giant tortoises, lava lizards, snakes and snails that infer San Cristóbal and Española as the starting point for these radiations [11,13,48,49]. In the present study, genomic data strongly support *P. leei* as the oldest extant species, suggesting that San Cristóbal was the first of the presently subaerial islands to be colonized. Our divergence time estimates across the *galapagensis* clade further support that *Phyllodactylus* geckos follow the island progression rule [10], as the age of species generally matches the age of the islands on which they occur (table 1), consistent with colonization proceeding from old to young islands.

The roles of dispersal and vicariance in the evolution of Galápagos leaf-toed geckos were discussed by Torres-Carvajal *et al*. [44], who proposed that some lineages might have speciated due to vicariance even though their species divergence time estimates predate the corresponding island emergence time estimates. Our lineage split estimates are slightly younger and provide better support for a vicariance speciation scenario for some species. The mean age estimate of the clade (hereafter central clade) including *P. galapagensis* (Santa Cruz), *P. duncanensis* (Pinzón), *P. andysabini* (northern Isabela), *P. simpsoni* (Isabela) and *P. maresi* (Santiago and Marchena) coincides with the 1.6 my estimate of a large central landmass, when the Galápagos archipelago started acting as a topographic barrier to the Equatorial undercurrent [50]. Similar to what has been proposed for tortoises [15], the diversification of leaf-toed geckos in the central clade was likely the result of dispersal across this large central landmass followed by vicariance episodes when this large landmass underwent fragmentation as some volcanoes started subsiding. Interestingly, the ADMIXTURE analysis of all species placed *P. andysabini*, *P. simpsoni* and *P. duncanensis* in the same cluster (electronic supplementary material, figure S5). However, a second ADMIXTURE analysis including only those three species placed *P. duncanensis* in a separate cluster, but failed to separate *P. andysabini* from *P. simpsoni* (electronic supplementary material, figure S6) despite all our samples of *P. simpsoni* being from extreme southern Isabela and the single sample of *P. andysabini* being from extreme northern Isabela (figure 1). These results suggest that *P. andysabini* and *P. simpsoni* could represent the same species.

Finally, the occurrence of *P. maresi* on Santiago and Marchena islands must have resulted from dispersal as Marchena is part of the Northern Galápagos Province; therefore, it has a separate origin and was never connected to the central islands [14,51]. Our divergence estimates for this dispersal event are relatively recent (approx. 600 ka) and the Marchena samples are nested within those from Santiago, supporting that dispersal proceeded from Santiago to Marchena. This is further supported by the ADMIXTURE analysis, which placed samples of *P. maresi* from Santiago and Marchena in the same cluster (electronic supplementary material, figure S5). Interestingly, the recently discovered Banco Tuzo seamount between Marchena and Galápagos was subaerial approximately 1.7 Ma and submerged approximately 350 ka [50,52]. Thus, Banco Tuzo may have served as a pathway for the dispersal of *P. maresi* from the Galápagos main platform (possibly Santiago) to Marchena, as has been suggested for tortoises and lava lizards [53].

### (b) No evidence of dispersal and introgression among Galápagos leaf-toed gecko taxa

Long distance dispersal is fundamental in generating terrestrial biodiversity on oceanic islands [54]. Among non-volant terrestrial vertebrates, lizards are the most successful island colonizers, capable of travelling long distances by passive drift (e.g. rafts of vegetation) on ocean currents [55–57]. Geckos are especially well adapted for sea travel as they have colonized remote islands [58] and even crossed the Atlantic ocean [59,60], although other groups like *Mabuya* lizards and even amphisbaenians have also succeeded in transatlantic rafting [61,62]. Leaf-toed geckos have been particularly successful in colonizing the Galápagos with three independent dispersals from South America, which suggests that they could easily move among islands within the archipelago. Indeed, leaf-toed geckos are present on 16 of the 19 islands larger than 1 km^2^, several of which are separated by water more than 200 m deep, indicating that geckos have successfully rafted between islands in the archipelago. Furthermore, retreating sea levels during Pleistocene glaciations reduced and even eliminated salt water barriers between some islands connected by land bridges, which is hypothesized to have facilitated inter-island dispersal of otherwise sea-locked terrestrial vertebrates [14,17]. Thus, it seems contradictory that our analyses failed to find any evidence of gene flow among *Phyllodactylus* species from different islands. A similar biogeographic pattern has been documented in other vertebrates, including Melanesian kingfishers and Lesser Sunda tree skinks, in which colonization and diversification across these archipelagos proceeded with no inter-island gene flow [63,64]. A proposed mechanism driving this pattern is a high fitness cost of inter-island migration, resulting in fewer offspring being produced by migrant individuals, ultimately leading to little or no gene flow between migrants and the resident island population [63].

Our results suggest that gene flow and hybridization between *Phyllodactylus* species are rare phenomena. Lack of gene flow or hybridization between species from different islands has also been suggested for other Galápagos terrestrial taxa that are capable of drifting on currents, such as lava lizards [13] and giant tortoises [65]. Furthermore, a recent single locus phylogeographic study of lava lizards and leaf-toed geckos from Floreana Island and two nearby (700 m and 8 km away) islets (Champion and Gardner, respectively) showed that populations on these three landmasses shared no haplotypes despite geographical proximity [66]. A similar result was obtained in another study on lava lizards from Santa Cruz Island and 12 close by islets [67]. How often lizards, tortoises and other ground-dwelling animals disperse among islands in the Galápagos archipelago is not known; however, rafting is probably rare because of thin tree coverage, sparse coastal vegetation coverage and lack of rivers. Further population genomic studies across a broader set of ground-dwelling species would clarify whether *Phyllodactylus* geckos represent the norm or an exception with respect to dispersal and gene flow across the archipelago.

Proposed factors limiting the successful invasion of migrants into established populations include behavioural barriers (e.g. similar to the proposed strong agonistic behaviour among divergent populations of giant tortoises), morphological specializations related to particular ecological resources and/or interspecific competition (e.g. saddleback tortoises in more arid habitats and dome shelled tortoises in more mesic habitats), and sexual selection [13,68,69]. Unfortunately, the ecology and behaviour of *Phyllodactylus* have not yet been extensively studied and thus it is unclear the extent to which species exhibit specialized adaptations to their local island habitats or whether they exhibit agonistic behaviour towards migrants. The single instance of *Phyllodactylus* sympatry in the Galápagos is among *P. leei* (the oldest extant member of the *galapagensis* clade) and *P. darwini* (the product of an independent dispersal that has not subsequently radiated), which co-occur on the island of San Cristóbal. Although the species can be found side by side in habitats across the island, they last shared a common ancestor approximately 19 million years ago and differ substantially in body size (*P. darwini* are up to approximately 60% larger in SVL [70,71]). Consequently, it is not surprising that we have not found evidence of hybridization between these distant relatives. Whether species of leaf-toed geckos in the *galapagensis* clade are reproductively isolated, however, remains an open question particularly given that the species are morphologically (and seemingly ecologically) similar. Previous population genetic studies of Galápagos geckos, lava lizards and mockingbirds distributed on small islets implicated a role for genetic drift in generating strong population differentiation over small geographic distances and short time scales [66,67,72,73]. Such shifts in allele frequencies could result in genetic incompatibilities and the evolution of postzygotic isolation (e.g. hybrid sterility and inviability [74]). Alternatively, leaf-toed geckos may have evolved prezygotic barriers to reproductive isolation. For instance, Zozaya *et al*. [75] demonstrated that pheromones in morphologically cryptic lineages of the Australian gecko *Heteronotia binoei* are lineage specific and have diverged among populations more so than morphology. Because pheromones influence behavioural isolation, pheromone divergence may lead to reproductive isolation even in morphologically similar taxa. Characterizing pheromone differentiation among species of Galápagos *Phyllodactylus* from different islands may be a promising future direction for assessing the role of behavioural isolation in the evolution of this group.

### (c) Asynchronous changes in population size pre-date the Last Glacial Maximum

Fluctuating sea levels throughout the Pleistocene have shifted the distribution and connectivity of terrestrial habitats on continental shelves and islands across the globe, causing shifts in species’ distributions and recurrent cycles of population connectivity and isolation [76]. Across the Galápagos archipelago, the islands are smaller at present than they were at the LGM [14,17] and thus we predicted that gecko populations across all islands would exhibit a recent and concurrent decrease in population size with cycles of population expansion and contraction mirroring glacial and interglacial episodes, respectively, in response to this shared palaeo-climatic history [77]. In contrast to our predictions, we inferred increases in population size in all species of the *galapagensis* clade prior to the LGM (expansion times from 20 to 150 ka), with three main periods of population expansion dating back to the Penultimate Glacial Period. Furthermore, we did not detect cyclical patterns of expansion and contraction mirroring climate-driven cycles of sea level rise and retreat throughout the Pleistocene. This lack of synchrony in population demography due to Quaternary climate cycles has been observed in several tropical forest systems [78], but information on how sea-level rise in particular may impact population demography in island systems is scarce [79]. Our results indicate that while sea-level fluctuations may impact population demography of island taxa, changes in the subaerial extent of a given island are not clear predictors of population size change and consequently that the mechanisms must be more nuanced. We also note that while we had small sample sizes for several of the taxa studied here (e.g. six haploid samples were used to construct the SFS in some species), these sampling schemes should be sufficient to discriminate among simple, single population models. However, simulation-based studies have demonstrated that larger sample sizes can increase the ability to correctly select demographic models and estimate parameters [80], and therefore future studies focused on more complex models of species diversification should increase sample sizes across the *galapagensis* clade.

Among the *galapagensis* clade species, we inferred low/intermediate synchrony in co-expansion in a global model including all species, indicating that historical population size changes were temporally heterogeneous. Additional nested analyses found three species exhibiting shared expansion in the Last Glacial Period and four species exhibiting shared expansion in the Penultimate Glacial Period (figure 3). There is no apparent relationship between the set of species exhibiting co-expansion and species age, island age or island size. This result is consistent with estimates of population demography in Galápagos tortoises, which also revealed a prevailing pattern of disparate histories that are seemingly not associated with species or island age [81]. We can only hypothesize that global climate cycles and sea level changes had different effects on habitat availability for geckos on each of the islands, leading to asynchronous co-expansion periods, and/or that biotic factors (e.g. competition, predation, habitat complexity) are also involved. Future efforts to characterize the natural history of Galápagos geckos would enable more explicit tests of both abiotic and biotic factors that may better explain the recent demographic history of this radiation. As more population genomic datasets become available for terrestrial species in the Galápagos and other archipelagos we will gain a better understanding of how global glacial cycles, and sea-level rise in particular, have or have not impacted the population demography of island communities.

## Data Availability

Sequence data are available on the NCBI Sequence Read Archive (BioProject number PRJNA1088269). Additional analyses and results supporting this study can be found in the electronic supplementary material [82] and on Dryad [83].
